# Cold water fish gelatin modification by a natural phenolic cross-linker (ferulic acid and caffeic acid)

**DOI:** 10.1002/fsn3.230

**Published:** 2015-04-24

**Authors:** Maryam Araghi, Zeinab Moslehi, Abdorreza Mohammadi Nafchi, Amir Mostahsan, Nima Salamat, Amir Daraei Garmakhany

**Affiliations:** 1 Food Biopolymer Research Group, Food Science and Technology Department, Damghan Branch, Islamic Azad UniversityDamghan, Semanan, Iran; 2 Department of Food Science and Technology, Toyserkan Faculty of Industrial Engineering, Bu-Ali Sina UniversityHamadan, Iran

**Keywords:** Caffeic acid, ferulic acid, oxygen permeability, solubility, vapor permeability

## Abstract

Nowadays use of edible films and coatings is increasing due to their biodegradability and environment friendly properties. Fish gelatin obtained from fish skin wastage can be used as an appropriate protein compound for replacing pork gelatin to produce edible film. In this study films were prepared by combination of fish gelatin and different concentration (0%, 1%, 3%, and 5%) of two phenolic compounds (caffeic acid and ferulic acid). The film was prepared at pH > 10 and temperature of 60˚c under continuous injection of O_2_ and addition of the plasticizer sorbitol/glycerol. Results showed that solubility, oxygen permeability, and water vapor permeability were decreased for caffeic acid and the highest effect was observed at concentration of 5%. Solubility had a linear relationship with concentration of phenolic compound in film containing ferulic acid, however, no significant change was observed in vapor and O_2_ permeability. A comparison between two phenolic compounds showed that caffeic acid had the highest effect in decreasing solubility, water vapor permeability, and oxygen permeability. Caffeic acid is more effective phenolic compound compared with Ferulic acid that can increase safety of biodegradable packaging by improving their barrier and physicochemical properties.

## Introduction

In recent years application of natural polymers for preparation of edible films aimed at packaging of food and pharmaceutical products has been of interest due to environmental and biodegradability properties (Cao et al. 2007; Bourtoom 2009; Zeppa et al. 2009). Proteins, Polysaccharides, lipids, and their derivatives are some examples of these polymer substances that used for production of edible films (Cao et al. 2007; Gómez-Guillén et al. 2009; Nawapat Detduangchan 2011; Voon et al. 2012). Although, synthetic films cannot completely replaced by these compounds, but their consumption could be reduce (Cao et al. 2007; Bourtoom 2009). Proteins are able to produce edible films with appropriate mechanical properties by formation of side chains via cross-linking (Huber et al. 2009). Gelatin is a protein obtained from collagen hydrolysis. Skin, bone, cartilage, and tandem of animals such as pork, fish, hide, and beef are some sources of gelatin extraction (Bigia et al. 2002; Cao et al. 2007; Kosaraju et al. 2010; Voon et al. 2012). Biodegradability, renewability (Zhang et al. 2010a,b), high productivity with low cost (de Carvalho and Grosso 2006), improved elasticity, consistency, and stability (Cao et al. 2007; Irwandi et al. 2009; See et al. 2010) of gelatin are mean reasons for it application in food and pharmaceutical industry (de Carvalho and Grosso 2006). Fish gelatin is an inexpensive compound that obtained from fish skin and bones residues (Sonthornvit and Krochta, 2000). It could be an appropriate alternative for pork gelatin that it consumption has been forbidden in Islam and Jew (Sonthornvit and Krochta, 2000; González et al., 2011). Also fish and fish gelatin are proper substitutes for red and white meat for vegetarian and there is no risk of bovine spongiform encephalopathy due to their consumption and trend to study these products have been increased (Sonthornvit and Krochta 2000*;* Bourtoom 2009*;* González et al. 2011). Fish gelatin has different behavior and characteristics compared to mammals' gelatin. Fish gelatin especially cold water fish gelatin has a high amount of hydrophobic amino acid and a little amounts of proline and hydroxy proline, and it has lower gelling ability as well as lower melting point than mammals gelatin (Sonthornvit and Krochta 2000; Sabato et al. 2001*;* Yi et al. 2006; Gómez-Guillén et al. 2009; González et al. 2011). Gelatin due to its lower hydrophobic and mechanical properties especially when exposed to moisture, shows low water vapor barrier properties and so due to this reason the use of this substance was limited (Yi et al. 2006; Cao et al. 2007*;* Wiwatwongwana and Pattana 2010; Zhang et al. 2010a; Bhat and Karim 2014). Structural modification may improve mechanical and barrier properties of gelatin. Several methods including physical (e.g., radiation treatments, ultrasound) and chemical (e.g., use of aldehydes, glutaraldehyde, and calcium salts) treatments, combination with other proteins and polysaccharides such as chitosan and casein, use of cross-link bounds forming compounds such as genipin, formaldehyde, transglutaminase enzyme, and natural plant products like phenolic compounds (e.g., caffeic acid, ferulic acid) were used for structural modification of gelatin (Hagiwara et al. 1991; Bor-Sen et al. 2008). In this study, two phenolic compounds which can form cross-link bounds namely caffeic acid and ferulic acid have been used for modification of cold water fish gelatin. Ferulic acid is an antioxidant, antimicrobial, anti cancer, and anti cholesterol factor which can react with some amino acids present at proteins such as tyrosine, lysine, and cysteine to form cross-link bounds (Cao et al. 2007). Caffeic acid is resulted from secondary metabolism of plant polyphenols and contains biochemical, antibacterial, and antiviral properties (Hagiwara et al. 1991). Although ferulic acid (Cao et al., 2007) and caffeic acid (Kosaraju et al., 2010) have been investigated for modification of bovine gelatin, there is no study regarding the effect of these compounds on cold water fish gelatin as well as on its barrier and physicochemical properties (Zeppa et al., 2009*;* ASTM standards 2005b).

## Material and Methods

### Materials

Gelatin from cold water fish (G7041-100G) was purchased from Sigma–Aldrich, Co (Kuala Lumpur, Malaysia, product of Canada). Food grade glycerol and liquid sorbitol were prepared in the laboratory grade. Caffeic acid (CA) and ferulic acid (FA) were obtained from Merck Company.

### Film preparation

The gelatin film-forming solutions were prepared by dissolving granules of cold water fish gelatin into deionized water to obtain a concentration of 5 g/50 mL (60°C for 1 h). Caffeic acid and ferulic acid were dispersed in 50 mL deionized water separately at different concentrations (1%, 3%, 5% w/v) and their pH was adjusted by sodium hydroxide 1 N (PH ≥ 10) and then heated (60°C) for 1 h under continuous stirring and injection of oxygen to produce a homogenized solution. After preparing of both solutions, they mixed (Cold water fish gelatin and two acids) and pH of produced mixture adjusted to pH ≥ 8 with sodium hydroxide 1 N and heated (60°C) for 30 min under continuous stirring and injection of oxygen to produce a homogenized solution. After completion of gelatinization, the solutions were cooled to room temperature. A portion (90 g) of the dispersion was cast on Perspex plates fitted with rims around the edge to yield a 16 × 16 cm^2^ film-forming area. The films were dried in an oven (40°C) for 20 h. Dried films were peeled and stored at 23 ± 2°C and 50 ± 5% relative humidity (RH) until examination.

### Thickness of film

The thickness of each film was measured at five different locations with a hand-held micrometer and accuracy of 0.01 mm (Mitutoyo, Tokyo, Japan).

### Film color measurement

For of color evolution of produced films a computer vision system (CVS) was used. The general methodology to convert RGB images into L*a*b* units is described by different scientists (Hashemi Shahraki et al. 2014; Mashkour et al. 2014). A brief description of each step follows:

1. Image acquisition: Images were captured using an image acquisition system for color digital camera similar to that developed (Papadakis et al. 2000) (Fig.1), namely:
  - Samples were illuminated using four fluorescent lamps (length of 60 cm) with a color temperature of 6500°k (Philips, Natural Daylight, 10W) and a color rendering index (Ra) close to 95%. The four lamps were arranged as a square 35 cm above the sample and at an angle of 45° with the sample plane to give a uniform light intensity over the food sample.
  - A color digital camera (CDC) Power Shot SX40 HS (Canon, Ota, Japan) was located vertically at a distance of 15 cm from the sample. The angle between the camera lens axis and the lighting sources was around 45°. The setting of the camera is given by Hashemi Shahraki et al. (2014).
  - Images were captured with the mentioned CDC at resolution (4000 × 3000 pixels) and connected to the USB port of a Pentium IV, the images directly in the computer in TIFF format without compression.

**Figure 1:**
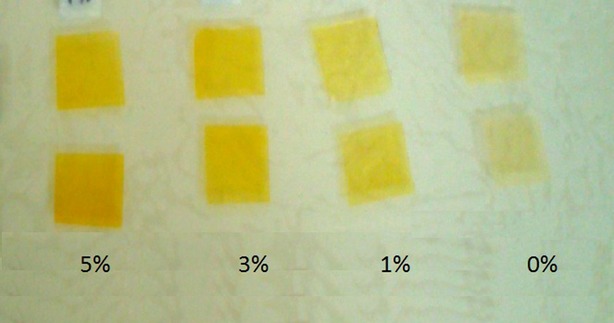
Effect of different concentrations of caffeic acid on color change of cold water fish gelatin films.
2. Image preprocessing: The digital images must be preprocessed to improve their quality before they are analyzed. Using digital filtering the noise of the image can be removed and the contrast can be enhanced.
3. Conversion of RGB images into L*a*b* units: Image J software (Version 4.4.Trial; NIH, Betheseda, MD, USA) used for analysis the image. In this stage the illumination standard had been applied.

### Water vapor permeability (WVP)

The modified gravimetric cup method based on ASTM E96-05 (ASTM 2005b) was used to determine the water vapor permeability (WVP) of films. The test cups were filled with 20 g of silica gel (desiccant) to produce a 0% RH below the film. The sample was placed between the cup and the ring cover of each cup coated with silicone sealant (high vacuum grease, Lithelin, Hannau, Germany). The air gap was at approximately 1.5 cm between the film surface and desiccant. The water vapor transmission rates (WVTR) of each film were measured at 55 ± 2% RH and 25 ± 2°C. The initial weight of the test cup was measured, and the cup was placed into an incubation chamber with an air velocity of 125 m/min. Weight gain measurements were taken by weighing the test cup with an electronic scale with accuracy of 0.0001 g (Sartorious Corp) every day for 7 days. A plot of weight gained versus time was used to determine the WVTR. The slope of the linear portion of this plot represented the steady state amount of water vapor diffusing through the film per time (g/h). The WVTR was expressed in term of g/m^2^ per day. Six samples per treatment were tested. The WVP of film was calculated by multiplying the steady WVTR in the film thickness and dividing them in to the water vapor pressure difference across the film.

### Water solubility of the films

Water solubility of the films was determined according to Maizura et al. (2007) and Laohakunjit and Noomhorm (2004) with some modifications. Pieces of film (2 × 3 cm^2^) were cut from each film and stored in a desiccators with P_2_O_5_ (0% RH) for 2 days. Samples were weighed and placed into beakers with 80 mL deionized water (18 MΩ). The samples were stirred with constant agitation for 1 h at room temperature. The remaining pieces of film after soaking were filtered through filter paper (Whatman no.1) and dried with hot oven (60°C) to reach constant weight. Samples were measured in triplicates and the percentage of total soluble matter (% solubility) was calculated as follow: 

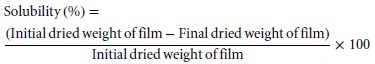
1

### Oxygen permeability (OP)

Oxygen permeability of films were measured by using the ASTM standard method D3985-05 and Mocon Oxtran 2/21 (Minneapolis, USA) machine equipped with a patented colometric sensor (Coulox®) and WinPermTM permeability software (ASTM 2005a). The films were placed on an aluminum foil mask with an open area of 5 cm^2^ and mounted in diffusion cells. Tests were carried out at 25°C, atmospheric pressure, and 50% RH using 21% oxygen as test gas. Transferred oxygen through the films was conducted by the carrier (N_2_/H_2_) gas to the colometric sensor. The permeability coefficients in cc-*μ*m/(m^2^ day atm) were calculated on the basis of oxygen transmission rate in steady state taking into account the films thickness.

### Statistical analysis

ANOVA and Tukey's post hoc tests were used for mean comparison of physical and mechanical properties of cold water fish gelatin films at the 5% significance level. Statistical analysis was conducted using GraphPad Prism 5 (GraphPad Software Inc., La Jolla, USA).

## Results and Discussion

### Film color

**Figure 2:**
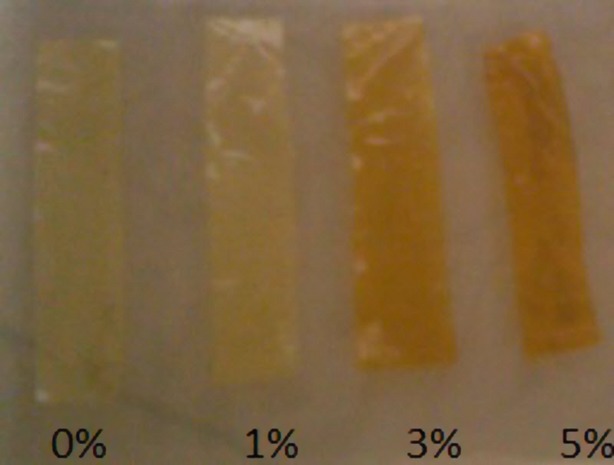
Effect of different concentrations of ferulic acid on color change of cold water fish gelatin films.

The color of produced films containing different concentrations of two phenolic compounds (Caffeic acid and ferulic acid) changed with variation in phenolic compounds concentration. Results showed that film color tends to be darker with the increase in phenolic compounds concentration. Films containing caffeic acid had darker color than films containing ferulic acid (Table1). In general interaction between natural phenolic compounds and proteins at presence of O_2_ and alkaline conditions leads to oxidation of phenolic structure and formation of quinon compound (Zhang et al. 2010a). In fact quinon is a dimmer compound which reacts with amino or sulfhidryl chain of polypeptide to form covalent bond of C-N or C-S. Polyphenol compounds are able to create cross-link bounds between individual protein molecules. The color change created in each phenolic compound shown at Figures 1 and 2 indicates oxidation of phenolic compounds. Zhang et al. (2010a,b) found a color change in bovine gelatin-based film containing caffeic acid from pale yellow to dark brown. These results are in agreement with ours (Zhang et al. 2010a).

**Table 1:** Effect of concentration and kind of phenolic compounds on the color of cold water fish gelatin

Phenolic compounds	Concentration	Color index			
		L^*^	a^*^	b^*^	ΔE
Caffeic acid	Blank or 0%	75.87^a^	1.06^a^	68.29^a^	–
	1%	78.58^a^	−5.31^b^	73.99^a^	8.97^b^
	3%	72.23^a^	−12.39^b^	64.02^a^	14.57^b^
	5%	65.11^b^	−13.52^b^	39.35^b^	34.14^a^
Ferulic acid	Blank or 0%	43.25^a^	1.78^c^	27.08^b^	–
	1%	46.45^a^	5.05^b^	32.35^b^	6.97^b^
	3%	41.72^ab^	13.45^a^	43.27^a^	20.02^a^
	5%	39.43^b^	14.80^a^	42.16^a^	20.29^a^

In each column and each phenolic compound, digits with same letter have no significant difference with each others (*P* > 0.05).

### Solubility

**Figure 3:**
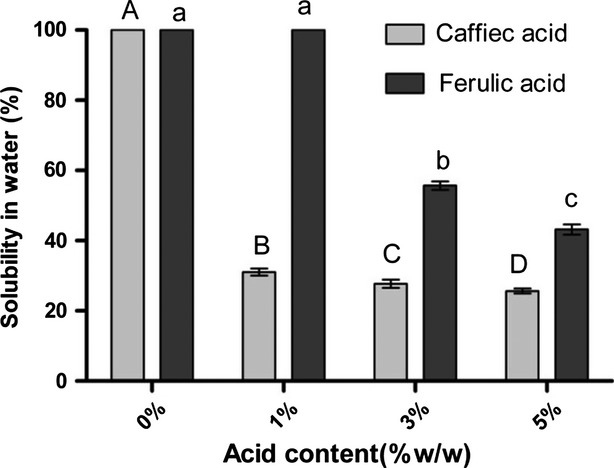
Solubility of fish gelatin films with phenolic compounds (caffeic acid (CA) and ferulic acid (FA)) added. In each phenolic compound, digits with same letter ( capital or small letters have no significant difference with each others (p>0.05).

Solubility of gelatin film of cold water fish modified by caffeic acid and ferulic acid is indicated at Figure 3. Results showed that increase in phenolic compounds concentration led to decrease in solubility of film. Results showed that caffeic acid (5%) due to formation of cross-linking between polymers led to the lowest solubility. Modification of fish gelatin by phenolic compounds led to reduced solubility which can be attributed to interaction of polymers by hydroxyl or carbonyl groups that lead to formation of hydrogen or covalent bonds, formation of cross-linking, and so reduction in water solubility of polymer. Reduction in solubility in fish gelatin film using ribose has been reported by Bahat et al. (2014). Zhang et al. (2010a,b) showed that Cross-linking gelatin with natural phenolic compound caffeic acid (CA) or tannic acid (TA) above pH 9 resulted in formation of insoluble hydrogels. The cross-linking reactivity was controlled by variation in pH, the concentration of the gelatin solution, or the amount of CA or TA used in the reaction.

### Oxygen permeability (OP)

**Figure 4:**
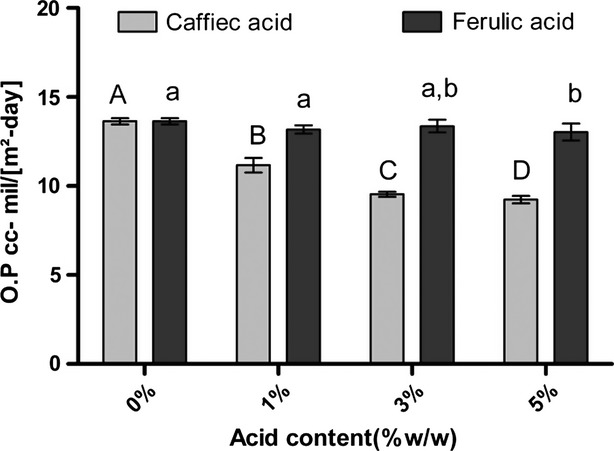
Oxygen permeability of fish gelatin films with phenolic compounds (caffeic acid (CA) and ferulic acid (FA)) added. In each phenolic compound, digits with same letter (capital or small letters) have no significant difference with each others (p>0.05)

Result of oxygen permeability is indicted in Figure4. Results showed that with the increase in phenolic compounds concentration oxygen permeability was decreased and caffeic acid had the lowest OP. Chemical nature of macromolecules, aggregation of molecules, and the amount of cross-linking are most factors that affect oxygen permeability of films. Generally, fish gelatin film has a low OP compared to gelatin film of other mammals (Yi et al. 2006). The amount of certain amino acids in protein can inhibit *α* helix formation and affect dynamic properties of gelatin (Irwandi et al. 2009). Proline and hydroxyl proline by formation of hydrogen bond enhanced firmness of *α*- helix structure. Fish gelatin shows a low OP due to low proline and hydroxyl proline content. Cross-link bound formation by phenolic compounds led to reduced OP as a result of increase caffeic acid concentration especially at concentration of 5%. Bor-Sen et al. (2008) investigated OP after modification of two fish gelatin by glutaraldehyde and found a decrease for Alaska Pollack species and an increase for Alaska pink Salmon.

### Water vapor permeability

Film diameter is an important factor influencing water vapor permeability. According to Fics low, water vapor permeability is decreased in thin layers films (<60 *μ*m diameter) (Huber et al. 2009). In this study no significant difference was found in film diameter. In films containing caffeic acid, increase in caffeic acid concentration led to decrease in water vapor permeability. However, in film containing ferulic acid no significant change was observed in water vapor permeability by increasing ferulic acid concentration and film containing ferulic acid had the lowest water vapor permeability. Generally, weak water vapor permeability of protein films limited their application in packaging (Patricia Yuca Hamaguchp and Munehiko 2003). Since water vapor permeability depends on the hydrophobic and hydrophilic components of film (Huber et al. 2009) and water vapor transfer is performed by hydrophilic component (Gómez-Guillén et al. 2009), cross-linking bound created by phenolic compounds (caffeic acid and ferulic acid) in cold water fish gelatin has led to reduction in water vapor permeability. Food packaging materials with appropriate barrier properties can improve packaging condition through lowering moisture transfer between food product and the environment (Voon et al. 2012). According to Table2 no significant change was observed in water vapor permeability of fish gelatin due to change in ferulic acid concentration. It can be attribute to high amounts of hydroxyl groups present in ferulic acid which can bound with water (Cao et al. 2007). Bhat and Karim (2014) reported a reduced water vapor permeability of fish gelatin film in which cross-linking has been created by ribose.

**Table 2:** Effect of phenolic compounds (CA, FA) concentration on the thickness and water vapor permeability of cold water fish gelatin

Concentration	Caffeic acid		Ferulic acid	
	Thickness	WVP	Thickness	WVP
0%	21.16 ± 0.94	7.52 ± 0.10	21.16 ± 0.94	7.52 ± 0.10
1%	19.93 ± 0.81	6.84 ± 0.15	20.70 ± 0.64	7.21 ± 0.27
3%	21.81 ± 0.61	6.55 ± 0.26	21.71 ± 0.72	7.47 ± 0.45
5%	21.68 ± 0.62	5.94 ± 0.23	20.11 ± 0.84	7.34 ± 0.20

## Conclusion

In this study, cold water fish gelatin was prepared from a combination of two phenolic compounds. Results showed that solubility, O_2_ permeability, and water vapor permeability have been reduced in gelatin film containing caffeic acid especially at concentration of 5%. In film containing ferulic acid, solubility was decreased by increase concentration but no significant change was observed in O_2_ permeability and water vapor permeability. With respect to importance of barrier and physicochemical properties for packaging products, caffeic acid is a more effective phenolic compound rather than ferulic acid that can increase safety of biodegradable packaging by improving it barrier and physicochemical properties.

## Conflict of Interest

None declared.
